# The Status of Occupational Burnout and Its Influence on the Psychological Health of Factory Workers and Miners in Wulumuqi, China

**DOI:** 10.1155/2020/6890186

**Published:** 2020-02-27

**Authors:** Yaoqin Lu, Zhe Zhang, Sunyujie Gao, Huan Yan, Lijiang Zhang, Jiwen Liu

**Affiliations:** ^1^Department of Occupational and Environmental Health, College of Public Health, Xinjiang Medical University, Wulumuqi, Xinjiang, China 830011; ^2^Department of Science and Education, Wulumuqi Center for Disease Control and Prevention, Wulumuqi, Xinjiang, China 830026; ^3^Xinjiang Engineering Technology Research Center for Green Processing of Nature Product Center, Xinjiang Autonomous Academy of Instrumental Analysis, Urumqi, Xinjiang, China 830011; ^4^Department of Occupational Disease Prevention and Control, Wulumuqi Center for Disease Control and Prevention, Wulumuqi, Xinjiang, China 830026

## Abstract

The purpose of this study was to investigate the status of occupational burnout and its influence on the psychological health of factory workers and miners, in order to provide theoretical basis and reference for alleviating occupational burnout and promoting psychological health. The cross-sectional study investigated 6130 factory workers and miners with online questionnaire; the Chinese Maslach Burnout Inventory (CMBI) and Symptom Check List-90 (SCL-90) were used. In total, 6120 valid questionnaires were collected; effectiveness was 99.8%. The percentage of the factory workers and miners suffering from occupational burnout was 85.98% and psychological health problems was 38.27%. A statistically significant difference was observed in relation to the prevalence of occupational burnout among factory workers and miners of different sex, education level, labor contracts, work schedule, monthly incomes, weight, hypertension, age, working years, working hours per day, working hours per week, coal dust, silica dust, asbestos dust, benzene, lead, and noise. The detection rate of psychological health was higher for males than females. The detection rate of psychological health was higher for working days per week less than 5 days than more than 5 days. The detection rate of psychological health with high school education, senior professional title, night shift, divorced, monthly income less than 3000 yuan, weight more than 75 kg, age more than 45 years, and working years between 25 and 30 years was higher than that of the other groups. The results showed that sex, education level, professional title, work schedule, monthly income, hypertension, age, working years, asbestos dust, benzene, and occupational burnout affected psychological health among factory workers and miners. Factory workers and miners had high levels of occupational burnout, and occupational burnout was a risk factor that can lead to psychological health.

## 1. Introduction

Occupational burnout refers to physical or mental exhaustion caused by overwork or stress [1]; it also can be described as a psychological syndrome characterized by exhaustion, cynicism, depersonalization, and reduced professional efficacy [2]. With the development of society and the increase of life pressure, people bear more and more pressure from society, work, and life. Occupational burnout has been regarded the crisis and illness in modern society and life.

Occupational stress, lifestyle, and personal relaxation have been shown to contribute to the development of occupational burnout and cause a series of psychological problems [3–5]. Previous literature review of studies in different occupational groups has indicated that classic risk factors such as high demands, low job control, high job strain, low reward, and job insecurity increased the risk for developing burnout [6]. Several studies have showed the effects of occupational burnout on psychological health, such as neurasthenia, anxiety disorder, and depression [7, 8]. But other surveys did not find the correlation between occupational burnout and psychological health [9, 10]. Thus, the relationship of occupational burnout and mental health needs to be further explored.

Factory workers and miners belong to a special occupational group who work in high-tension conditions, and a demanding work environment with dust, chemical factors, physical factors, and biological factors can detrimentally affect employees' psychological health, leading to job stress and burnout [11]. There is a lack of research about the association between occupational burnout and individual characteristics or occupational hazards of factory workers and miners. Therefore, this study administered a questionnaire survey to factory workers and miners in Wulumuqi, China, to investigate the status of occupational burnout and its influence on psychological health, in order to provide theoretical basis and reference for alleviating occupational burnout and promoting psychological health.

## 2. Materials and Methods

### 2.1. Participants

This survey was carried out from January to May 2019. Workers on the occupational exposures of coal dust, silica dust, asbestos dust, benzene, lead, noise, and Brucella in factories and mines in Urumqi, China, were investigated. A total of 6500 factory workers and miners were initially selected using a cluster sampling method. Participants without the occupational exposures according to their working environment were excluded. Those with work experience less than one year or taking psychoactive drugs were also excluded. According to the inclusion and exclusion criteria, 6130 participants were included in this survey. The cross-sectional study was conducted by online questionnaire using a mobile phone. The respondents volunteered to participate in the survey, and the written informed consent was provided. Finally, 6120 questionnaires were collected and 10 copies of continuous answer questionnaires were excluded. The effectiveness was 99.8%.

### 2.2. Chinese Maslach Burnout Inventory (CMBI)

The Chinese Maslach Burnout Inventory (CMBI) was established by Li Yongxin which was based on Maslach Burnout Inventory (MBI). The Cronbach *α* for the CMBI was 0.88, split-half reliability coefficient was 0.84, and KMO was 0.91, respectively. The CMBI consisted of 15 items about three dimensions: emotional exhaustion, depersonalization, and reduced personal accomplishment; subjects responded to each item ranging from 1 (completely fitting) to 7 (completely unfitting). According to the critical values (emotional exhaustion ≥ 25, depersonalization ≥ 11, and reduced personal accomplishment ≥ 16), occupational burnout was divided into four levels: none (subjects' scores on three factors were lower than the critical value), mild (subjects' scores on any one factor were equal to or higher than the critical value), moderate (subjects' scores on any two factors were equal to or higher than the critical value), and severe (subjects' scores on three factors were equal to or higher than the critical value) [12–15].

### 2.3. Symptom Check List-90 (SCL-90)

The Symptom Check List-90 (SCL-90) was established by L.R. Derogatis in 1975 and widely used in psychiatric outpatient examination because of its high authenticity in evaluating various mental health surveys [16, 17]. The Cronbach *α* for the SCL-90 was 0.99, split-half reliability coefficient was 0.98, and KMO was 0.99, respectively. There were 9 dimensions (including 90 items) in SCL-90, and each item was assigned a score ranging from 1 (not have) to 5 (serious). The 9 dimensions were somatization, obsessive-compulsive symptoms, interpersonal sensitivity, depression, anxiety, hostility, phobia, paranoid ideation, and psychosis. The higher score showed a worse psychological symptom. The result was positive, and further examination was needed, when the total score was more than 160, or any item score was more than 2, or the number of positive items was more than 43 [18].

### 2.4. Quality Control

All the investigators were trained before the survey. In order to ensure the completeness of the online questionnaire, each item was set as required. If there was any missing value, the questionnaire cannot be submitted. The validity analysis of the data was completed by senior data analysts.

We facilitated the preinvestigation before the formal investigate, in order to train investigators and foster cooperation. We contacted face-to-face interviews with each participant to complete the online questionnaire and solve their concerns timely.

### 2.5. Statistical Methods

The results were analyzed by R software (Version: 3.5.2). A chi-squared test was used for the counting data; multiple logistic regression analysis was used to estimate the relationship between multiple factors. The significance level (*α*) was set at 0.05.

## 3. Results

### 3.1. General Demographic Characteristics of Factory Workers and Miners

Among the 6120 workers and miners, 4017 were men (65.64%) and 2103 were women (34.36%); 1220 had hypertension (19.93%) and 364 had diabetes (5.95%). Exposure to coal dust, silica dust, asbestos dust, benzene dust, lead, noise, and brucellosis accounted for 1446 (23.63%), 622 (10.16%), 935 (15.28%), 1947 (31.81%), 353 (5.77%), 4545 (74.26%), and 108 (1.76%), respectively (Table 1).

### 3.2. Comparison of Occupational Burnout Levels in Different Populations

The survey results showed that 85.98% of workers and miners experienced occupational burnout in varying degrees. There were statistically significant differences in sex (*P* < 0.001), education level (*P* < 0.001), labor contracts (*P* < 0.001), work schedule (*P* < 0.001), monthly incomes (*P* = 0.019), weight (*P* < 0.001), hypertension (*P* < 0.001), age (*P* < 0.001), working years (*P* < 0.001), working hours per day (*P* < 0.001), working hours per week (*P* = 0.001), coal dust (*P* < 0.001), silica dust (*P* < 0.001), asbestos dust (*P* < 0.001), benzene (*P* < 0.001), lead (*P* = 0.003), and noise (*P* < 0.05) (Table 2).

### 3.3. Comparison of Psychological Health in Different Populations

The results showed that the detection rate of psychological health was higher for males than females (*P* = 0.003). The detection rate of psychological health was higher for working days per week less than 5 days than more than 5 days (*P* = 0.029). The detection rate of psychological health with high school education (*P* < 0.001), senior professional title (*P* < 0.001), night shift (*P* < 0.001), divorced (*P* < 0.001), monthly income less than 3000 yuan (*P* < 0.001), weight more than 75 kg (*P* < 0.001), age more than 45 years (*P* < 0.001), and working years between 25 and 30 years (*P* < 0.001) was higher than that of the other groups. The psychological health was related to the workers and miners who had diabetes (*P* < 0.001), hypertension (*P* < 0.001), and exposure to coal dust (*P* < 0.001), silica dust (*P* < 0.001), asbestos dust (*P* < 0.001), benzene (*P* < 0.001), lead (*P* < 0.001), and noise (*P* < 0.001) (Table 3).

### 3.4. Exploration of Factors Influencing Psychological Health

Multiple logistic regression analysis was used to analyze the effects of different characteristics and occupational burnout on the psychological health of factory workers and miners. All the independent variables in the logistic regression were stratified. The results showed that education level of junior college and higher (*P* < 0.001), work schedule of shift and day and night shift (*P* < 0.001), monthly income (except for 7000~) (*P* < 0.005), hypertension (*P* < 0.001), working years (*P* < 0.005), asbestos dust (*P* < 0.001), benzene (*P* = 0.021), and occupational burnout (*P* < 0.001) affected psychological health of factory workers and miners. Higher education, shift work or day and night shift, lower income, hypertension, longer working years, exposure to asbestos dust and benzene, and occupational burnout were risk factors related to poorer psychological health (Table 4).

## 4. Discussion

Occupational burnout is a state of pressure that is a significant issue worldwide which is related to efficiency and quality of work, and it is also regarded as particularly harmful to the social psychological of the working population [19, 20]. A study conducted by Inger et al. examined the occupational burnout of southern Sweden teachers and found that 46.8% teachers suffered from burnout [21]. Guan et al. found that the rate of occupational burnout among civil servants was 45.0% [22]. A survey in China had revealed that the prevalence of occupational burnout in the military was 88.14% [23, 24]. While occupational burnout can affect physical and psychological health, it also adversely impacts upon the working ability and quality.

Factory workers and miners belong to a special professional group, whose mental health is closely related to the development of the industry. However, the workers and miners' social status is low, and they work hard but the income is relatively low [19]. Long periods of heavy work caused them to languish and burnout. And they often worked in a special environment of high temperature, high pressure, darkness, or dust; some studies already proved that people living in harsh environments have a higher risk of developing mental illnesses, and the special environments affect the degree of job burnout [25–28]. Our research presented here revealed that 85.98% of factory workers and miners experience occupational burnout, reminding that occupational burnout is prevalent among this particular working group. The higher the level of occupational burnout, the poorer the psychological health of factory workers and miners, suggesting that occupational burnout is a risk factor that can influence psychological health.

This survey investigated occupational burnout levels among factory workers and miners. The occupational burnout level of night shift workers was higher than that of others, which may due to long-term working at night causing night and day reversal and lack of rest, thereby resulting in fatigue. Chronic diseases such as hypertension could cause changes in the body's functioning that can make workers feel more tired at work. People under 30 years old or with less than 10 working years were more likely to develop occupational burnout. Most of them had acquired professional skills and had good stamina so that they were more eager to seek promotion opportunity or to increase their personal income [29]. Workers who worked more than 7 hours per day or more than 5 days per week need to maintain a high level of stress, and lack of time of recreation, leisure, and relaxation increased their burnout levels, which might enhance the risk of mental health problems [30, 31]. Long-term occupational exposure to coal dust, silica dust, asbestos dust, benzene, lead, and noise would cause varying degrees of pulmonary diseases and other illness, thereby affecting respiration and body metabolism, which makes them prone to fatigue and tired.

The study found that education level had influence on psychological health; the risks of psychological health problems at junior college and bachelor's degree or above were 1.80 times and 2.03 times that of junior high school and below, respectively. Maslach' study had showed that people who had higher education may have more self-expectation and social expectation [32]. When the job cannot meet one's personal needs and expectation, one may experience strain response such as job satisfaction drops, occupational burnout, and mental illness [33–35]. Work schedule was a risk factor related to poor psychological health, particularly at shift and day and night shift. The risk of psychological health problems increased with changing a way of work schedule and/or of day and night shifts was the highest. The long-term day and night shifts made workers' day and night reversed, resulting in the different physical functions and thereby leading to mental illness. Khajehnasiri et al.'s research also showed that shift workers had a high level of stress and depressive symptoms [36]. The influence of marital status on psychological health was statistically significant in univariate analysis, but not in multiple logistic regression analysis, which meant marital status was not an independent risk factor of psychological health. But some studies confirmed the correlation of divorce and psychological problems due to lack of a sense of family and kinship [37, 38]. Due to the poor physical health, workers with hypertension were liable to suffer from cardiovascular diseases and thereby have some psychological changes, which was consistent with other studies [39]. The influence of occupational burnout at any level on psychological health was statistically significant, and the risks of psychological health problems increased 1.43 times, 3.82 times, and 25.53 times with aggravating occupational burnout level, respectively. According to psychological theories, excess psychological stress could decline psychological function (such as distracted attention and reduced working will and desire) and cause negative physiological responses (such as declined strength, stiffened body, and disorders in sense and memory) [40]. The higher the occupational burnout, the more significant the adverse physiological function and psychological reaction, leading to increasing the possibility of work errors. When workers and miners can no longer utilize their internal and social resources to relieve their psychological burden caused by work errors, their psychological balance will be disturbed, resulting in emotional fluctuations and psychological health problems [41].

It reminded that reasonable arrangement of work shift, promotion of occupational personal protection and health education, guidance to spare time arrangement of workers, enhancement of disease prevention, and psychological counseling should be taken into consideration to keep physical and mental health of factory workers and miners.

The present survey used online questionnaire; compared with paper questionnaire, the recovery rate was higher, but there were still repeated answers and cross-sectional investigation cannot establish a causal relationship between diseases; in the future, further studies will continue to explore the relationship between the factors and diseases by using cohort studies.

## 5. Conclusion

In conclusion, this survey found that the factory workers and miners generally suffered from occupational burnout, and sex, education level, professional title, work schedule, monthly income, hypertension, age, working years, asbestos dust, and benzene were related risk factors. In addition, occupational burnout influenced the psychological health. Measures need to be taken to ease occupational burnout among factory workers and miners in order to improve their psychological health.

## Figures and Tables

**Table 1:** Characteristics of the factory workers and miners.

Items	Groups	Case number	Percentage (%)
Sex	Male	4017	65.64
	Female	2103	34.36

Ethnicity	Han	5016	81.96
	Other	1104	18.04

Education level	Junior high school and below	652	10.65
	High school	1227	20.05
	Junior college	2722	44.48
	Bachelor's degree or above	1519	24.82

Labor contracts	Signed	5896	96.34
	Unsigned	224	3.66

Professional title	No	2349	38.38
	Primary	1326	21.67
	Middle	1483	24.23
	Senior	962	15.72

Work schedule	Day shift	3289	53.74
	Night shift	201	3.28
	Shift	1897	31.00
	Day and night shifts	733	11.98

Marital status	Unmarried	857	14.00
	Married	4864	79.48
	Divorced	357	5.83
	Widowed	42	0.69

Monthly income (yuan)	<3000	1656	27.06
	3000~	2093	34.20
	4000~	1329	21.72
	5000~	659	10.77
	6000~	205	3.35
	7000~	86	1.41
	8000~	92	1.50

Weight (kg)	<55	840	13.73
	55~	1571	25.67
	65~	1780	29.08
	75~	1929	31.52

Chronic disease	Diabetes	364	5.95
	Hypertension	1220	19.93

Age (years)	<25	319	5.21
	25~	634	10.36
	30~	790	12.91
	35~	704	11.50
	40~	723	11.81
	45~	2950	48.20

Working years (years)	~5	920	15.03
	5~	831	13.58
	10~	840	13.73
	15~	319	5.21
	20~	695	11.36
	25~	1266	20.69
	30~	1249	20.41

Working hours per day (hours)	≤7	975	15.93
	>7	5145	84.07

Working days per week (days)	≤5	4006	65.46
	>5	2114	34.54

Occupational hazard factors	Coal dust	1446	23.63
	Silica dust	622	10.16
	Asbestos dust	935	15.28
	Benzene	1947	31.81
	Lead	353	5.77
	Noise	4545	74.26
	Brucellosis	108	1.76

**Table 2:** Comparison of occupational burnout levels in different populations.

Items	Groups	CMBI				CMBI detection rate (%)	Chi-squared value	*P* value
		No	Mild	Moderate	Severe			
Sex	Male	548	1371	1699	399	0.86	24.078	**<0.001**
	Female	310	831	785	177	0.85		

Ethnicity	Han	700	1794	2042	480	0.86	1.274	0.735
	Other	158	408	442	96	0.86		

Education level	Junior high school and below	68	346	209	29	0.90	121.637	**<0.001**
	High school	144	433	519	131	0.88		
	Junior college	386	926	1162	248	0.86		
	Bachelor's degree or above	260	497	594	168	0.83		

Labor contracts	Signed	839	2069	2417	571	0.86	59.719	**<0.001**
	Unsigned	19	133	67	5	0.86		

Professional title	No	334	868	934	213	0.86	9.941	0.355
	Primary	183	488	540	115	0.85		
	Middle	201	507	610	165	0.86		
	Senior	140	339	400	83	0.86		

Work schedule	Day shift	524	1252	1267	246	0.84	69.783	**<0.001**
	Night shift	19	64	91	27	0.91		
	Shift	235	620	813	229	0.88		
	Day and night shifts	80	266	313	74	0.89		

Marital status	Unmarried	120	342	333	62	0.86	14.988	0.091
	Married	683	1715	1986	480	0.86		
	Divorced	51	125	152	29	0.86		
	Widowed	4	20	13	5	0.90		

Monthly income (yuan)	<3000	218	598	686	154	0.87	32.453	**0.019**
	3000~	271	725	892	205	0.87		
	4000~	190	499	512	128	0.86		
	5000~	118	225	256	60	0.82		
	6000~	34	72	81	18	0.83		
	7000~	16	40	23	7	0.81		
	8000~	11	43	34	4	0.88		

Weight (kg)	<55	118	371	298	53	0.86	57.312	**<0.001**
	55~	211	610	616	134	0.87		
	65~	253	596	763	168	0.86		
	75~	276	625	807	221	0.86		

Diabetes	Yes	50	122	148	44	0.90	3.621	0.305
	No	808	2080	2336	532	0.86		

Hypertension	Yes	148	360	539	173	0.88	63.275	**<0.001**
	No	710	1842	1945	403	0.86		

Age (years)	<25	39	152	117	11	0.88	57.433	**<0.001**
	25~	97	261	242	34	0.85		
	30~	102	270	334	84	0.87		
	35~	112	232	279	81	0.84		
	40~	105	259	285	74	0.85		
	45~	403	1028	1227	292	0.86		

Working years (years)	~5	138	448	295	39	0.85	133.982	**<0.001**
	5~	112	315	339	65	0.87		
	10~	103	284	358	95	0.88		
	15~	53	106	127	33	0.83		
	20~	91	239	287	78	0.87		
	25~	163	425	521	157	0.87		
	30~	198	385	557	109	0.84		

Working hours per day (hours)	≤7	179	339	384	73	0.82	21.028	**<0.001**
	>7	679	1863	2100	503	0.87		

Working days per week (days)	≤5	606	1383	1641	376	0.85	17.405	**0.001**
	>5	252	819	843	200	0.88		

Coal dust	Yes	181	476	624	165	0.87	19.090	**<0.001**
	No	677	1726	1860	411	0.86		

Silica dust	Yes	55	208	275	84	0.87	29.043	**<0.001**
	No	803	1994	2209	492	0.86		

Asbestos dust	Yes	105	268	414	148	0.91	74.537	**<0.001**
	No	753	1934	2070	428	0.85		

Benzene	Yes	237	592	856	262	0.89	89.269	**<0.001**
	No	621	1610	1628	314	0.85		

Lead	Yes	37	108	163	45	0.88	13.676	**0.003**
	No	821	2094	2321	531	0.85		

Noise	Yes	590	1553	1935	467	0.87	60.824	**<0.001**
	No	268	649	549	109	0.83		

Brucellosis	Yes	14	42	37	15	0.87	3.772	0.287
	No	844	2160	2447	561	0.86		

**Table 3:** Comparison of psychological health in different populations.

Items	Groups	SCL-90		SCL detection rate (%)	Chi-squared value	*P* value
		-	+			
Sex	Male	2426	1591	39.61	8.70	**0.003**
	Female	1352	751	35.71		

Ethnicity	Han	3088	1928	38.44	0.30	0.585
	Other	690	414	37.50		

Education level	Junior high school and below	516	136	20.86	93.95	**<0.001**
	High school	727	500	40.75		
	Junior college	1620	1102	40.48		
	Bachelor's degree or above	915	604	39.76		

Professional title	No	1514	835	35.55	45.01	**<0.001**
	Primary	873	453	34.16		
	Middle	867	616	41.54		
	Senior	524	438	45.53		

Work schedule	Day shift	2159	1130	34.36	46.62	**<0.001**
	Night shift	111	90	44.78		
	Shift	1093	804	42.38		
	Day and night shifts	415	318	43.38		

Marital status	Unmarried	624	233	27.19	54.31	**<0.001**
	Married	2928	1936	39.80		
	Divorced	200	157	43.98		
	Widowed	26	16	38.10		

Monthly income (yuan)	<3000	970	686	41.43	45.52	**<0.001**
	3000~	1234	859	41.04		
	4000~	846	483	36.34		
	5000~	458	201	30.50		
	6000~	142	63	30.73		
	7000~	61	25	29.07		
	8000~	67	25	27.17		

Weight (kg)	<55	581	259	30.83	49.11	**<0.001**
	55~	1016	555	35.33		
	65~	1094	686	38.54		
	75~	1087	842	43.65		

Diabetes	Yes	186	178	48.90	18.05	**<0.001**
	No	3592	2164	37.60		

Hypertension	Yes	557	663	54.34	165.85	**<0.001**
	No	3221	1679	34.27		

Age (years)	<25	257	62	19.44	136.33	**<0.001**
	25~	463	171	26.97		
	30~	537	253	32.03		
	35~	425	279	39.63		
	40~	443	280	38.73		
	45~	1653	1297	43.97		

Working years (years)	~5	746	174	18.91	267.53	**<0.001**
	5~	575	256	30.81		
	10~	540	300	35.71		
	15~	196	123	38.56		
	20~	385	310	44.60		
	25~	636	630	49.76		
	30~	700	549	43.96		

Working hours per day (hours)	≤7	599	376	38.56	0.03	0.864
	>7	3179	1966	38.21		

Working days per week (days)	≤5	2433	1573	39.27	4.77	**0.029**
	>5	1345	769	36.38		

Coal dust	Yes	809	637	44.05	26.50	**<0.001**
	No	2969	1705	36.48		

Silica dust	Yes	307	315	50.64	44.30	**<0.001**
	No	3471	2027	36.87		

Asbestos dust	Yes	427	508	54.33	119.75	**<0.001**
	No	3351	1834	35.37		

Benzene	Yes	999	948	48.69	130.65	**<0.001**
	No	2779	1394	33.41		

Lead	Yes	177	176	49.86	20.79	**<0.001**
	No	3601	2166	37.56		

Noise	Yes	2698	1847	40.64	41.61	**<0.001**
	No	1080	495	31.43		

Brucellosis	Yes	57	51	47.22	3.36	0.067
	No	3721	2291	38.11		

**Table 4:** Effects of psychological health-related factors among workers and miners according to the results of the multiple logistic regression analysis.

Variable	Groups	*β* (95% CI)	S.E.	OR (95% CI)	Wald	*P* value
Intercept		-3.76 (-4.34, -3.18)	0.30	0.02 (0.01, 0.04)	-12.709	0.000

Sex	Male	—	—	—	—	—
	Female	0.09 (-0.05, 0.23)	0.07	1.09 (0.95, 1.26)	1.206	0.228

Ethnicity	Han	—	—	—	—	—
	Other	0.02 (-0.14, 0.17)	0.08	1.02 (0.87, 1.19)	0.213	0.831

Education level	Junior high school and below	—	—	—	—	—
	High school	0.26 (-0.00, 0.52)	0.13	1.30 (1.00, 1.69)	1.943	0.052
	Junior college	0.59 (0.34, 0.83)	0.13	1.80 (1.41, 2.30)	4.668	**0.000**
	Bachelor's degree or above	0.71 (0.45, 0.98)	0.14	2.03 (1.56, 2.66)	5.245	**0.000**

Professional title	No	—	—	—	—	—
	Primary	0.08 (-0.09, 0.25)	0.09	1.08 (0.92, 1.29)	0.968	0.333
	Middle	0.13 (-0.03, 0.29)	0.08	1.14 (0.97, 1.33)	1.563	0.118
	Senior	0.17 (-0.01, 0.35)	0.09	1.19 (0.99, 1.42)	1.891	0.059

Work schedule	Day shift	—	—	—	—	—
	Night shift	0.31 (-0.03, 0.65)	0.17	1.36 (0.97, 1.91)	1.782	0.075
	Shift	0.32 (0.17, 0.47)	0.07	1.38 (1.19, 1.59)	4.312	**0.000**
	Day and night shifts	0.42 (0.23, 0.62)	0.10	1.52 (1.26, 1.86)	4.270	**0.000**

Marital status	Unmarried	—	—	—	—	—
	Married	0.07 (-0.17, 0.30)	0.12	1.07 (0.85, 1.35)	0.565	0.572
	Divorced	0.16 (-0.17, 0.49)	0.17	1.17 (0.85, 1.64)	0.971	0.332
	Widowed	-0.02 (-0.76, 0.72)	0.38	0.98 (0.47, 2.06)	-0.051	0.960

Monthly income (yuan)	<3000	—	—	—	—	—
	3000~	-0.18 (-0.33, -0.03)	0.08	0.84 (0.72, 0.97)	-2.305	**0.021**
	4000~	-0.30 (-0.48, -0.12)	0.09	0.74 (0.62, 0.89)	-3.272	**0.001**
	5000~	-0.58 (-0.82, -0.34)	0.12	0.56 (0.44, 0.71)	-4.831	**0.000**
	6000~	-0.55 (-0.92, -0.18)	0.19	0.58 (0.40, 0.84)	-2.917	**0.004**
	7000~	-0.44 (-1.00, 0.11)	0.28	0.64 (0.37, 1.12)	-1.566	0.117
	8000~	-0.59 (-1.11, -0.06)	0.27	0.55 (0.33, 0.94)	-2.189	**0.029**

Diabetes	No	—	—	—	—	—
	Yes	0.52 (0.37, 0.67)	0.08	1.23 (0.96, 1.58)	1.638	0.101

Hypertension	No	—	—	—	—	—
	Yes	0.21 (-0.04, 0.46)	0.13	1.68 (1.44, 1.96)	6.656	**0.000**

Age (years)	<25	—	—	—	—	—
	25~	0.14 (-0.25, 0.53)	0.20	1.15 (0.78, 1.69)	0.714	0.475
	30~	-0.01 (-0.43, 0.41)	0.21	0.99 (0.65, 1.51)	-0.045	0.964
	35~	0.26 (-0.17, 0.70)	0.22	1.30 (0.84, 2.01)	1.181	0.238
	40~	0.09 (-0.36, 0.53)	0.23	1.09 (0.70, 1.71)	0.376	0.707
	45~	0.20 (-0.25, 0.64)	0.22	1.22 (0.78, 1.89)	0.870	0.384

Working years (years)	~5	—	—	—	—	—
	5~	0.34 (0.08, 0.61)	0.14	1.40 (1.08, 1.84)	2.517	**0.012**
	10~	0.37 (0.08, 0.66)	0.15	1.45 (1.08, 1.93)	2.483	**0.013**
	15~	0.51 (0.15, 0.88)	0.19	1.67 (1.16, 2.41)	2.764	**0.006**
	20~	0.79 (0.47, 1.12)	0.17	2.20 (1.59, 3.06)	4.768	**0.000**
	25~	0.87 (0.54, 1.19)	0.17	2.39 (1.72, 3.30)	5.239	**0.000**
	30~	0.68 (0.36, 1.01)	0.17	1.97 (1.43, 2.75)	4.093	**0.000**

Working hours per day (hours)	≤7	—	—	—	—	—
	>7	0.07 (-0.10, 0.25)	0.09	1.07 (0.90, 1.28)	0.834	0.404

Working days per week (days)	≤5	—	—	—	—	—
	>5	0.13 (-0.01, 0.26)	0.07	1.14 (0.99, 1.29)	1.877	0.060

Coal dust	No	—	—	—	—	—
	Yes	0.12 (-0.03, 0.27)	0.08	1.13 (0.97, 1.31)	1.589	0.112

Silica dust	No	—	—	—	—	—
	Yes	0.19 (-0.02, 0.39)	0.11	1.21 (0.98, 1.48)	1.747	0.081

Asbestos dust	No	—	—	—	—	—
	Yes	0.39 (0.21, 0.57)	0.09	1.48 (1.23, 1.77)	4.186	**0.000**

Benzene	No	—	—	—	—	—
	Yes	0.16 (0.02, 0.30)	0.07	1.17 (1.02, 1.34)	2.311	**0.021**

Lead	No	—	—	—	—	—
	Yes	-0.15 (-0.42, 0.12)	0.14	0.86 (0.66, 1.12)	-1.108	0.268

Noise	No	—	—	—	—	—
	Yes	0.13 (-0.02, 0.27)	0.07	1.14 (0.98, 1.31)	1.751	0.080

Brucellosis	No	—	—	—	—	—
	Yes	0.26 (-0.21, 0.72)	0.24	1.30 (0.81, 2.05)	1.077	0.281

CMBI	None	—	—	—	—	—
	Mild	0.36 (0.15, 0.56)	0.11	1.43 (1.16, 1.75)	3.373	**0.001**
	Moderate	1.34 (1.14, 1.53)	0.10	3.82 (3.12, 4.63)	13.282	**0.000**
	Severe	3.24 (2.93, 3.54)	0.16	25.53 (18.80, 34.63)	20.774	**0.000**

## Data Availability

The data used to support the findings of this study are available from the corresponding author upon request.

## References

[1] Ofei-Dodoo S., Kellerman R., Gilchrist K., Casey E. M. (2019). Burnout and quality of life among active member physicians of the medical society of Sedgwick County. *Kansas Journal of Medicine*.

[2] Maslach C., Leiter M. P. (1997). *The Truth about Burnout: How Organizations Cause Personal Stress and What to Do about It*.

[3] Gluschkoff K., Elovainio M., Kinnunen U. (2016). Work stress, poor recovery and burnout in teachers. *Occupational Medicine*.

[4] Håkansson C., Ahlborg G. (2017). Occupational imbalance and the role of perceived stress in predicting stress-related disorders. *Scandinavian Journal of Occupational Therapy*.

[5] Lindegård A., Jonsdottir I. H., Börjesson M., Lindwall M., Gerber M. (2015). Changes in mental health in compliers and noncompliers with physical activity recommendations in patients with stress-related exhaustion. *BMC Psychiatry*.

[6] Aronsson G., Theorell T., Grape T. (2017). A systematic review including meta-analysis of work environment and burnout symptoms. *BMC Public Health*.

[7] Bianchi R., Laurent E., Schonfeld I. S., Verkuilen J., Berna C. (2018). Interpretation bias toward ambiguous information in burnout and depression. *Personality and Individual Differences*.

[8] Hartley T. A., Violanti J. M., Sarkisian K. (2014). Association between police-specific stressors and sleep quality: influence of coping and depressive symptoms. *Journal of Law Enforcement Leadership Ethics*.

[9] Ruddock S., Rahimi-Golkhanden S., Ruddock-Hudson M., Wollersheim D. (2019). Tracking the mental health outcomes of occupational burnout with Australian Rules Football coaches: a 2-year longitudinal study. *Journal of Science and Medicine in Sport*.

[10] Salas M. L., Quezada S., Basagoitia A. (2015). Working conditions, workplace violence, and psychological distress in Andean miners: a cross-sectional study across three countries. *Annals of Global Health*.

[11] Parent-Thirion A., Biletta I., Cabrita J., Llave V. O., Vermeylen G., Wilczynska A. (2017). *6th European Working Conditions Survey: Overview Report*.

[12] Freudenberger H. J. (1974). Staff burnout. *Journal of Social Issues*.

[13] Li Y. X., Wu M. Z. (2005). A structural study of job burnout. *Psychological Science*.

[14] Li F. Y., Liu J. W., Lian Y. L. (2009). The reliability and validity analysis of the tool for measuring mental workers’ job burnout. *China Journal of Occupational Health and Labor*.

[15] Maslach C., Jackson S., Leiter M. (1996). *MBI: Maslach Burnout Inventory Manual*.

[16] Christensen J., Fisker A., Mortensen E. L. (2015). Comparison of mental distress in patients with low back pain and a population-based control group measured by Symptoms Check List - a case-referent study. *Scandinavian Journal of Public Health*.

[17] Bech P., Bille J., Møller S. B., Hellström L. C., Østergaard S. D. (2014). Psychometric validation of the Hopkins Symptom Checklist (SCL-90) subscales for depression, anxiety, and interpersonal sensitivity. *Journal of Affective Disorders*.

[18] Leão I. A. T., Del Porto J. A. (2012). Cross validation with the mood disorder questionnaire (MDQ) of an instrument for the detection of hypomania in Brazil: The 32 item hypomania symptom check- list, first Revision (HCI-32-R_1_). *Journal of Affective Disorders*.

[19] Li F. Y., Tao N., Xing R. (2010). Analysis on status and influential factors of job burnout of police. *China Occupational Medicine*.

[20] Chen H., Wu P., Wei W. (2012). New perspective on job burnout: exploring the root cause beyond general antecedents’ analysis. *Psychological Reports*.

[21] Arvidsson I., Leo U., Larsson A., Håkansson C., Persson R., Björk J. (2019). Burnout among school teachers: quantitative and qualitative results from a follow-up study in southern Sweden. *BMC Public Health*.

[22] Guan S., Xiaerfuding X., Ning L. (2017). Effect of job strain on job burnout, mental fatigue and chronic diseases among civil servants in the Xinjiang Uygur Autonomous Region of China. *International Journal of Environmental Research and Public Health*.

[23] Lei J., Yi J. H., Wu S. W. (2009). Analysis on professional burnout of teachers in certain newly upgraded college. *Shanghai Journal of Prevention Medicine*.

[24] Li Y. X., Li Y. M. (2007). Relationship among job burnout, self-esteem, health and intention to quit of nurses. *Chinese Journal of Nursing*.

[25] Danhof-Pont M. B., van Veen T., Zitman F. G. (2011). Biomarkers in burnout: a systematic review. *Journal of Psychosomatic Research*.

[26] Slobodskaya H. R., Akhmetova O. A., Ryabichenko T. I. (2007). Siberian child and adolescent mental health: prevalence estimates and psychosocial factors. *Alaska Medicine*.

[27] Tao N., Zhang J., Song Z., Tang J., Liu J. (2015). Relationship between job burnout and neuroendocrine indicators in soldiers in the Xinjiang arid desert: a cross-sectional study. *International Journal of Environmental Research and Public Health*.

[28] Mänty M., Kouvonen A., Lallukka T., Lahti J., Lahelma E., Rahkonen O. (2015). Changes in working conditions and physical health functioning among midlife and ageing employees. *Scandinavian Journal of Work, Environment & Health*.

[29] Li Y., Sun X., Ge H., Liu J., Chen L. (2019). The status of occupational stress and its influence the quality of life of copper-nickel miners in Xinjiang, China. *International Journal of Environmental Research and Public Health*.

[30] Wall T. D., Bolden R. I., Borrill C. S. (1997). Minor psychiatric disorder in NHS trust staff: occupational and gender differences. *The British Journal of Psychiatry : the Journal of Mental Science*.

[31] Fu A., Liu B., Jiang Y., Zhao J., Zhang G., Liu J. (2017). A mental health survey of different ethnic and occupational groups in Xinjiang, China. *International Journal of Environmental Research and Public Health*.

[32] Ning L., Guan S., Liu J. (2018). An investigation into psychological stress and its determinants in Xinjiang desert oil workers. *Medicine*.

[33] Maslach C., Schaufeli W. B., Leiter M. P. (2001). Job burnout. *Annual Review of Psychology*.

[34] Chickering A. W., Dalton J. C., Stamm L. (2015). *Encouraging Authenticity and Spirituality in Higher Education*.

[35] Nabirye R. C., Brown K. C., Pryor E. R., Maples E. H. (2011). Occupational stress, job satisfaction and job performance among hospital nurses in Kampala, Uganda. *Journal of Nursing Management*.

[36] Khajehnasiri F., Mortazavi S. B., Allameh A., Akhondzadeh S., Hashemi H. (2013). Total antioxidant capacity and malondialdehyde in depressive rotational shift workers. *Journal of Environmental and Public Health*.

[37] Beseler C. L., Stallones L. (2008). A cohort study of pesticide poisoning and depression in Colorado farm residents. *Annals of Epidemiology*.

[38] Blümel J. E., Chedraui P., Baron G. (2009). Sexual dysfunction in middle-aged women. *Menopause*.

[39] Yu H., Liu J. C., Fan Y. J. (2016). Association between occupational stressors and type 2 diabetes among Chinese police officers: a 4-year follow-up study in Tianjin, China. *International Archives of Occupational and Environmental Health*.

[40] Poitras V. J., Pyke K. E. (2013). The impact of acute mental stress on vascular endothelial function: evidence, mechanisms and importance. *International Journal of Psychophysiology*.

[41] Zhang W. (2014). Causation mechanism of coal miners' human errors in the perspective of life events. *International Journal of Mining Science and Technology*.

